# Comparison of commercial RNA extraction kits for preparation of DNA-free total RNA from *Salmonella *cells

**DOI:** 10.1186/1756-0500-3-211

**Published:** 2010-07-27

**Authors:** Lydia V Rump, Benedicta Asamoah, Narjol Gonzalez-Escalona

**Affiliations:** 1 Center for Food Safety and Applied Nutrition, Food and Drug Administration, 5100 Paint Branch Parkway, College Park, MD 20740, USA

## Abstract

**Background:**

The isolation of DNA-free RNA is a crucial step in the reverse transcription PCR (RT-PCR). Every RNA extraction procedure results in RNA samples contaminated with genomic DNA, which can cause false-positive outcomes in highly sensitive applications, including a recently developed quantitative real-time PCR (RT-qPCR) assay that targets *inv*A mRNA for the detection of live *Salmonella *cells. The assay of this specific mRNA can be used to indicate the presence of live, as opposed to dead, cells of *Salmonella enterica *in a food matrix.

**Findings:**

We evaluated the ability of five RNA extraction kits to produce RNA preparations from exponentially growing *Salmonella *cells. The acceptability of the preparations for use in downstream applications such as RT-qPCR was judged in terms of the total amount of RNA recovered, the integrity of the RNA molecules, and minimal content of DNA. The five kits produced RNA preparations that differed markedly in yield, integrity of the *Salmonella *RNA and the amount of contaminant DNA. The greatest RNA recovery was achieved with the MasterPure kit; however, the preparation contained high levels of genomic DNA. The UltraClean extraction kit gave a low level of RNA recovery with a poor level of integrity. The RNeasy Mini, RiboPure and PureLink extraction kits produced high-quality, DNA-free RNA suitable for *Salmonella *detection by RT-qPCR.

**Conclusions:**

We showed that the RNeasy Mini and PureLink RNA extraction kits were the most suitable for the detection of *Salmonella invA *mRNA by RT-qPCR. The use of these two kits will greatly reduce the frequency of false-positive results and might allow fast RT-qPCR determination of *invA *mRNA produced by viable *Salmonella *in food samples.

## Background

Commercially available RNA extraction kits are rapid, capable of high-throughput analysis and cost-effective [1]. The isolation of DNA-free RNA is crucial to the success of highly sensitive assays like RT-PCR. RNA extraction procedures frequently result in RNA preparations that are highly contaminated with genomic DNA, which often leads to false-positive RT-PCR outcomes. The presence of DNA in an RNA sample can be detected easily by an appropriate PCR test of an indicator gene. Then, if necessary, treatment of the RNA preparation with DNase I will usually eliminate, or at least substantially reduce, the content of DNA [2].

The RNA concentration of a sample is commonly determined via measurement of absorbance at a wavelength of 260 nm (*A*_260_). The purity of the RNA sample can be determined using the *A*_260_/*A*_280 _ratio as a reference (a value of ~2.0 is considered "pure" RNA). However, the accuracy of this method is questionable, because protein contamination can cause an overestimation (>50%) of RNA content [3,4]. Moreover, RNA is susceptible to degradation by RNases present in the sample, which can result in shorter fragments of RNA and this decrease in RNA integrity might interfere with downstream applications; e.g. microarray expression profiles [5]. Another technique commonly used to determine the concentration and extent of degradation of an RNA sample is agarose gel electrophoresis with subsequent banding pattern analysis [4]. However, this approach relies on the human eye and is prone to errors of interpretation.

There are methods that allow for the accurate estimation of RNA concentration. Modern spectrometric methods, such as spectrophotofluorimetry (Nanodrop ND-3000, Fisher Scientific), in combination with RNA RiboGreen dye (Molecular Probes, Invitrogen) can be used for ultrasensitive RNA quantification http://www.nanodrop.com/Library/art-gen-state-microsample-quantitation.pdf. Lab-on-chip technology, such as the Agilent 2100 Bioanalyzer (Agilent technologies) and Experion (Bio-Rad Laboratories) are widely used to estimate RNA quality and quantity [6]. Agilent has developed software to calculate the RNA integrity number (RIN), a qualitative assessment of RNA quality [5]. RIN values range from 1 to 10, with 1 being the most degraded and 10 the least degraded [6].

The present study evaluated the performance of five commonly used commercial RNA extraction kits for isolating cellular RNA, using actively growing *Salmonella *SE5 as a model organism. The kits evaluated were: the RiboPure Bacteria Kit (Ambion), the PureLink RNA Mini Kit (Invitrogen), the UltraClean Microbial RNA Isolation Kit (MoBio, Carlsbad, CA), the RNeasy Mini Kit (Qiagen) and the MasterPure RNA Purification Kit (EPICENTRE). The kits were compared for (1) the yield of RNA, (2) the polymeric length integrity of the RNA and (3) the amount of DNA present in the RNA preparation.

## Methods

### Bacterial strains and media

*Salmonella enterica *serovar Enteritidis strain SE5 was grown overnight in Luria-Bertani (LB) medium_at 35°C with shaking (250 rpm).

### Nucleic acid extraction

The performance of five commercial RNA extraction kits was evaluated: RiboPure-Bacteria Kit (Ambion, Inc), PureLink RNA Mini Kit (Invitrogen), UltraClean Microbial RNA Isolation Kit (MoBio), RNeasy Mini Kit (QIAGEN) and MasterPure RNA Purification Kit (EPICENTRE Biotechnologies). The principles for RNA purification of these kits are very similar. The UltraClean and the RiboPure kits use a bead cell disruption system. Four of the kits, except the MasterPure kit, use spin-column technology and selective binding properties of silica membranes. The RNA adsorbs to the silica membrane in the presence of high concentrations of salt. Contaminants are unable to bind to the silica column and therefore pass through the column. The loaded column is washed and then any bound RNA is eluted. The MasterPure kit uses a salt-precipitation protocol, instead of a column, to purify the RNA and it captures the small RNA molecules that tend to be lost when using columns. All RNA extractions were done in triplicate (1 ml each) with exponentially growing SE5 cells and following the manufacturer's recommendations for each kit. The final RNA fraction was obtained by elution or suspended in 50 μl of DEPC-treated water (Ambion). The treatment with DNase I (Invitrogen) was done at 37°C for 30 min and the DNase was inactivated by incubation at 65°C for 10 min.

### Determination of RNA concentration and RNA integrity number (RIN)

The RNA concentration in individual RNA samples was determined using the RNA Pico 6000 LabChip kit (Agilent Technologies). The LabChips were run in an Agilent 2100 Bioanalyzer following the manufacturer's instructions (Agilent). The use of the RNA Pico 6000 LabChip kit allowed determination of the RNA integrity number (RIN), an indicator of the integrity of the RNA in the sample, using 2100 Bioanalyzer Expert software [5].

### Determination of DNA contamination in the RNA samples before and after treatment with DNase I

The *invA *mRNA levels were measured by RT-qPCR as described [7]. The *invA *RT-qPCR reactions were done as described [7], but without an internal control. Briefly, RT-qPCR reactions were done with the SuperScriptTM III Platinum One-Step Quantitative RT-PCR System kit essentially according to the manufacturer's instructions (Invitrogen) but with reactions scaled down to a final volume of 20 μl and MgCl_2 _added to the master mix to a final concentration of 5 mM. The final concentration of each primer was 200 nM: (invA_176F 5'-CAACGTTTCCTGCGGTACTGT-3') and invA_291R (5'-CCCGAACGTGGCGATAATT-3'). The final concentration of the probe invA_Tx_208 was 150 nM (5'-TX-CTCTTTCGTCTGGCATTATCGATCAGTACCA-BHQ2-3'). RT-qPCR and data analysis (in triplicate) were done with a Rotor-Gene 3000 (Corbett) real-time PCR instrument using 2 μl of each RNA sample. Additionally, each RNA sample was amplified by *invA *qPCR to detect DNA contamination and to estimate the number of *invA *DNA copies present in the sample. The RT-qPCR conditions were as follows: 15 min at 50°C for the generation of the cDNAs, 2 min at 95°C to activate the hot-start Taq polymerase and then 40 cycles of denaturation at 95°C for 15 s, and primer annealing and extension at 60°C for 30 s (the acquisition of Texas Red emission was performed at the end of this cycle). Identical conditions were used for qPCR reactions. The reported efficiency of the qPCR and RT-qPCR for these primers is 0.93 - 0.99 and 0.90 - 0.96, respectively [7]. The term Cq is equivalent to the original Ct (threshold cycle) terminology [8]. A difference between the Cq values for RT-qPCR and qPCR of >4 cycles was considered a positive result. Differences in the range 3.1 - 3.6 cycles between samples are mostly due to differences in concentration of about 10-fold (if efficiency is 90 - 110%) https://www.genomics.agilent.com/CalculatorPopupWindow.aspx?CalID=8. For Cq values that dipped below this range, the RNA sample was considered inadequate for the detection of *Salmonella invA *mRNA.

## Results and discussion

### Determination of RNA concentration and RIN values

Total RNA concentrations were determined with a 2100 Bioanalyzer and the RNA 6000 Pico LabChip kit (Agilent) [1]. As expected, the highest mean level of RNA (1.82 ± 0.36 μg/μl) was obtained using The MasterPure kit, which does not include passage through a column and, therefore, is not subject to limitations imposed by column binding capacity. The RNA yield from column-based extraction methods depends greatly on the binding capacity of the column, which is designed primarily for small-scale RNA extractions, where this limitation has little impact. Next in order of RNA recovery were the column-based PureLink (0.97 ± 0.32 μg/μl), RNeasy (0.78 ± 0.32 μg/μl) and RiboPure (0.28 ± 0.05 μg/μl) kits. The smallest RNA yield (0.05 ± 0.03 μg/μl) was obtained with the UltraClean kit. This descending order of recovery of the column-based kits parallels the descending order of binding capacity stated by the manufacturers: PureLink (up to 1000 μg), RNeasy (100 μg), RiboPure (90 μg) and UltraClean (60 μg).

The PureLink and the RNeasy kits use chemical methods to lyse the cells, whereas the RiboPure and UltraClean kits physically disrupt the bacterial cell walls by beating the cells with beads. Greater disruption would be expected to improve the RNA yield; however, the bead-based kits yielded less RNA than the others. This suggests that the different RNA yields of these kits are not due only to different extents of cell lysis.

RNA molecules are thermodynamically stable but can be digested rapidly by RNases present in the cell lysate, which could result in short RNA fragments that can compromise downstream applications [5]. RIN values for each RNA sample were obtained with a 2100 Bioanalyzer (Agilent) and an RNA 6000 Pico LabChip kit. Most of the kits extracted largely intact *Salmonella *RNA, as indicated by the RIN values of 8 or higher (Table 1). The exception was the UltraClean kit, which yielded RNA of lower integrity with a mean RIN value of 5.65 ± 4.03 (Table 1). Because of the influence of RNA integrity on downstream applications, samples with RIN values <8 might have a negative influence on the outcome of an experiment. However, an RNA sample could be degraded to an extent that precluded a genome-wide microarray experiment but might still deliver good RT-PCR data.

**Table 1:** Mean RNA integrity number (RIN) of total RNA extracted from exponentially growing *Salmonella *SE5 cells by 5 kits

RNA extraction kit	Manufacturer	RIN
RiboPure-Bacteria Kit	Ambion	9.30 ± 0.36
PureLink RNA Mini Kit	Invitrogen	9.07 ± 1.62
RNeasy Mini Kit	QIAGEN	9.57 ± 0.59
MasterPure RNA Purification Kit	EPICENTRE Biotechnologies	8.00 ± 1.21
UltraClean Microbial RNA Isolation Kit	Mo Bio	5.65 ± 4.03

### RT-qPCR analysis (invA mRNA)

RT-PCR and RT-qPCR are important tools for the detection of pathogens in foods [7,9-11]. The RT-qPCR assay depends on the use of intact RNA; the higher the RNA quality, the lower the variability of the results [12]. In this study, we determined the influence of the degree of RNA integrity on the RT-qPCR mRNA detection and quantification in RNA samples extracted from exponentially growing *Salmonella *[7]. An RT-qPCR assay of *invA *mRNA in tandem with qPCR of *invA *DNA was used to detect the presence of DNA in the RNA preparations produced by five RNA extraction kits and to assess the extent of its degradation by treatment with DNase I. The DNA polymerase is incapable of amplifying RNA, so the qPCR (*invA *DNA) results indicate the presence of DNA in the RNA samples [13].

The *invA *mRNA RT-qPCR and *invA *qPCR results are given in Tables 2 and 3, respectively, for the RNA samples obtained with the kits before and after treatment with DNase I. All of the kits yielded RNA samples containing large amounts of DNA (Table 2). After treatment with DNase I, the DNA content of the RNA samples obtained with four of the kits was reduced to levels that did not interfere with the detection of *invA *mRNA (Table 3). Therefore, treatment with DNase I is absolutely required for eliminating all traces of DNA. In the case of the highest initial level of DNA contamination observed with the MasterPure kit, some traces of DNA remaining in the sample could interfere with RT-qPCR assays and so this kit was excluded from the evaluation of the effect of RIN values on the RT-qPCR results.

**Table 2:** Inability of RT-qPCR to detect *invA *mRNA in RNA extracts not treated with DNase I [[Bibr B7]]

RNA extraction kit	Manufacturer	*invA *mRNA copies/rxn **RT-qPCR [Cq]**^**a**^	*invA *DNA copies/rxn **qPCR [Cq]**^**b**^	*invA *mRNA presence **(Cq qPCR - Cq RT-qPCR)**^**c**^
RiboPure-Bacteria	Ambion	(1.9 ± 1.6) × 10^6 ^[25.5 ± 1.8]	(1.8 ± 1.8) × 10^6 ^[24.8 ± 1.4]	-0. 7 (Negative)
PureLink RNA Mini	Invitrogen	(1.2 ± 0.5) × 10^8 ^[18.8 ± 0.6]	(1.5 ± 0.8) × 10^8 ^[18.5 ± 0.7]	-0.3 (Negative)
RNeasy Mini	QIAGEN	(7.9 ± 2.2) × 10^7 ^[19.3 ± 0.4]	(1.1 ± 0.3) × 10^8 ^[18.9 ± 0.4]	-0.4 (Negative)
MasterPure RNA Purification	EPICENTRE Biotechnologies	(5.8 ± 0.8) × 10^8 ^[16.4 ± 0.2]	(8.8 ± 0.9) × 10^8 ^[15.9 ± 0.2]	-0.3 (Negative)
UltraClean Microbial RNA Isolation	MoBio	(2.2 ± 0.6) × 10^8 ^[17.8 ± 0.4]	(3.4 ± 0.8) × 10^8 ^[17.3 ± 0.3]	-0.5 (Negative)

rxn, reaction.

^*a*^*invA *mRNA copies per reaction are mean values of 6 determinations ± standard deviations.

^*b*^*invA *DNA copies per reaction are mean values of 6 determinations ± standard deviations.

^c^- If <4 is negative or inconclusive for *invA *mRNA presence due to DNA contamination.

Detection limit of the *invA *qRT-PCR and *invA *qPCR are 40 and 10 copies per reaction, respectively [7]

**Table 3:** Effect of DNase I treatment of RNA extracts on ability to detect *invA *mRNA by RT-qPCR

RNA extraction kit	Manufacturer	*invA *mRNA copies/rxn **RT-qPCR [Cq]**^**a**^	*invA *DNA copies/rxn **qPCR [Cq]**^**b**^	*invA *mRNA presence **(Cq qPCR - Cq RT-qPCR)**^**c**^
RiboPure-Bacteria	Ambion	(5.46 ± 0.88) × 10^5 ^[26.5 ± 0.9]	<10 [37.1 ± 1.5]	10.6 (Positive)
PureLink RNA Mini	Invitrogen	(7.71 ± 1.89) × 10^5 ^[26.0 ± 1.2]	<10 [37.5 ± 1.8]	11.5 (Positive)
RNeasy Mini	QIAGEN	(7.82 ± 1.25) × 10^6 ^[21.5 ± 0.5]	<10 [36.5 ± 1.7]	15 (Positive)
MasterPure RNA Purification	EPICENTRE Biotechnologies	(1.19 ± 2.38) × 10^8 ^[18.7 ± 1.0]	(3.10 ± 2.60) × 10^7 ^[21.5 ± 1.2]	2.8 (Negative)
UltraClean Microbial RNA Isolation	MoBio	(1.28 ± 1.12) × 10^5 ^[28.6 ± 0.6]	(1.00 ± 1.20) × 10^3 ^[33.2 ± 1.5]	4.6 (Positive)

rxn, reaction.

^*a*^*invA *mRNA copies per reaction are mean values of 6 determinations ± standard deviations.

^*b*^*invA *DNA copies per reaction are mean values of 6 determinations ± standard deviations.

^c^- If <4 is negative or inconclusive for *invA *mRNA presence due to DNA contamination.

Detection limit of the *invA *qRT-PCR and *invA *qPCR are 40 and 10 copies per reaction, respectively [7].

We observed that the RIN value of a *Salmonella *RNA sample does not appear to be correlated with the ability to detect *invA *mRNA by RT-qPCR. The RNA samples produced by the UltraClean kit had the lowest RIN value, indicating a high level of RNA degradation, and contained fewer *invA *mRNA copies than the RNA samples produced by the other kits (Table 2). However, these numbers of *invA *mRNA copies might be due to the low initial concentration of RNA in the sample (0.05 ± 0.03 μg/μl) and not to the quality of the RNA sample, which has been observed by others for different types of RNA samples [14]. The concentrations of RNA in the samples produced by the UltraClean kit were about 5 - 19-fold lower than those produced by the other kits. Accordingly, the amount of *invA *mRNA molecules in that sample ought to be smaller and could explain the 4 - 11-fold fewer copies observed for this kit compared to the others (Table 3). In any case, due to its low yield of total RNA and its low RIN value, the UltraClean kit is unsuitable for applications such as the detection of *invA *mRNA by RT-qPCR. The RiboPure, PureLink and RNeasy kits are the most suitable for sensitive RT-qPCR assays, such as the detection of *invA *mRNA in *Salmonella*, because they yielded high average recovery levels of RNA with high RIN values and low levels of DNA.

## Conclusion

Overall, this comparison showed that most of the kits tested were suitable for *Salmonella *RNA extraction. The greatest concentration of total RNA was obtained with the MasterPure kit; however, the sample contained high levels of genomic DNA, which interferes with *invA *mRNA detection by RT-qPCR. Therefore, the MasterPure kit could be most useful for assays, such as northern blot analysis, that require large amounts of RNA and are unaffected by the presence of DNA. The RIN values obtained with the RiboPure, PureLink, RNeasy and MasterPure extraction kits were within the acceptable range for RNA integrity. The UltraClean kit isolated highly degraded RNA (RIN value <6), which is unsuitable for some RNA assays. As expected, the treatment of RNA samples with DNase I after extraction appears to be absolutely required to reduce the amount of residual DNA, especially for sensitive assays like *invA *RT-qPCR. The RNeasy and RiboPure extraction kits produced large yields of RNA with a high degree of integrity and could be considered the kits of choice for the detection of *Salmonella invA *mRNA by RT-qPCR.

## Competing interests

The authors declare that they have no competing interests.

## Authors' contributions

LVR did most of the RNA extractions, participated in the experimental design, and production of the draft of the manuscript. BA did some RNA extractions; participated in the experimental design, and production of the draft of the manuscript. NGE conceived the study, participated in the experimental design and coordination, did the RT-qPCR assays, and participated in production of the draft of the manuscript. All authors have read and approved the final manuscript.
